# Activation of Silenced Cytokine Gene Promoters by the Synergistic Effect of TBP-TALE and VP64-TALE Activators

**DOI:** 10.1371/journal.pone.0095790

**Published:** 2014-04-22

**Authors:** Kim Anthony, Abhijit More, Xiaoliu Zhang

**Affiliations:** 1 Department of Biology and Biochemistry, University of Houston, Houston, Texas, United States of America; 2 Center for Nuclear Receptors and Cell Signaling, University of Houston, Houston, Texas, United States of America; Texas A&M University, United States of America

## Abstract

Recent work has shown that the combinatorial use of multiple TALE activators can selectively activate certain cellular genes in inaccessible chromatin regions. In this study, we aimed to interrogate the activation potential of TALEs upon transcriptionally silenced immune genes in the context of non-immune cells. We designed a unique strategy, in which a single TALE fused to the TATA-box binding protein (TBP-TALE) is coupled with multiple VP64-TALE activators. We found that our strategy is significantly more potent than multiple TALE activators alone in activating expression of IL-2 and GM-CSF in diverse cell origins in which both genes are otherwise completely silenced. Chromatin analysis revealed that the gene activation was due in part to displacement of a distinctly positioned nucleosome. These studies provide a novel epigenetic mechanism for artificial gene induction and have important implications for targeted cancer immunotherapy, DNA vaccine development, as well as rational design of TALE activators.

## Introduction

Transcription Activator-Like Effectors (TALE) technology comprises a rapidly developing tool for targeted genome manipulation. Deciphering of the TALE DNA recognition code in 2009 led to the development of a series of novel engineered TALE chimeras for a variety of purposes [1], [2]. For example, TALEs have been engineered to modulate gene expression [3]–[10], reprogram epigenetic modifications [11], [12], repair or disrupt genes using TALENs or chimeric TALE recombinases [13], [14], and promote targeted gene transposition using TALE-directed piggyBac [15]. TALEs offer an attractive advantage over traditional zinc finger-based technologies due to their inherently simple and predictable DNA recognition code attributed to its novel type DNA-binding domain (DBD). The TALE DBD is highly repetitive and contains a tandem array of repeat monomers (typically 15.5–19.5 repeats) with each monomer consisting of ∼34 amino acids with the exception of the last repeat unit consisting of ∼20 amino acids [1], [2]. Each repeat binds a single DNA base pair coordinated at the 12^th^ and 13^th^ amino acid positions of each repeat monomer specifically known as the **r**epeat **v**ariable **d**i-residue (RVD), which has the following deciphered code: NI = A, HD = C, NG = T, NN = G/A [1], [2]. In contrast to zinc finger proteins, DNA recognition by TALE repeat monomers has no apparent sequence context requirements [3], [16], [17] and exhibits far less cellular toxicity than zinc fingers [18], [19]. To date, TALE technology has been applied to a broad range of model organisms [13]. Hence, TALE technology provides a well-established and versatile platform to develop genome engineering tools that are important for interrogation of functional genomics in model organisms, development of novel gene therapies and innovative synthetic biology tools.

Relative to TALENs, the potential of TALEs as activators has not been fully explored until more recently. Initial studies showed that a single TALE activator was able to drive the expression of a reporter gene linked to a synthetic promoter derived from different cellular genes [3], [5]. However, in contrast to synthetic promoters, TALE-mediated activation of endogenous genes was only moderate and in some instances, completely failed to activate gene expression [5]–[7], [10]. Further analysis found that the activation of an epigenetically silenced gene required a combination of epigenetic modifiers acting together with a TALE activator, while a single TALE was unable to stimulate such a combinatorial effect and was thus inefficient in activating the silenced Oct4 promoter [8]. Recently, two studies have demonstrated that this limitation can be overcome by targeting multiple TALE activators to a gene promoter for synergistic gene activation [20], [21]. Furthermore, these studies reveal that targeting of TALE activators to open chromatin regions within gene promoters are not a requirement for successful gene activation, suggesting that TALEs can override repressive chromatin structures through cooperative binding to a gene promoter [21]. However, some key elements in the promoter regions, such as the TATA-box and transcription initiation site, have not been fully evaluated in the context of TALE technology for gene activation purposes.

In eukaryotic cells, initiation of transcription begins with the recognition and binding of promoter-specific transcriptional activators to their cognate DNA response elements within a gene promoter. An activator functions as a platform to recruit and assemble chromatin remodelers and components of the basal transcriptional machinery [22]. More specifically, activators recruit the TATA-box binding protein (TBP) to gene promoters, resulting in the formation of the preinitiation complex (PIC) comprised of TBP-associated factors (TAFs), transcription factor II proteins (e.g. TFIIB), and RNA Pol II (RNAP) to stimulate mRNA transcription [23]. Consequently, TBP binding to the TATA box is the rate limiting step in transcriptional initiation [24]. Hence, we rationed that targeted recruitment of TBP using a linked TALE DNA-binding domain (DBD) to a particular TATA box could lead to transcriptional initiation of the selected gene by bypassing this crucial rate-limiting step. Several studies have shown that artificial recruitment of non-classical activators (e.g. TBP) coupled with classical activators (e.g. VP16) can synergize gene expression [25]–[27]. To date, no study has examined the utility of such a strategy using TALE activators for targeted activation of silent gene expression. Therefore, we aimed to interrogate TALE activation potential utilizing the combined action of chimeric TBP and VP64-TALE activators applied to a classical example of gene silencing observed within T-cell biology illustrated by IL-2 and GM-CSF genes.

Our data shows that TALE fused to TBP (TBP-TALE) acts synergistically with other VP64-TALE activators and this combination is significantly more efficacious than multiple TALE activators alone in activating expression of IL-2 and GM-CSF in diverse non-immune cells in which both genes are otherwise completely silenced. Chromatin analysis revealed that the gene activation was due in part to displacement of a distinctly positioned nucleosome. These studies provide a novel epigenetic mechanism for artificial gene induction, and have important implications for cancer immunotherapy, DNA vaccine development, as well as rational design of TALE activators.

## Materials and Methods

### Construction of TALEs and Target Site Selection

TALE plasmids were obtained from Addgene (http://www.addgene.org/TALeffector/goldengateV2) and constructed as previously described [28]. Briefly, the corresponding TALE DNA-binding domain was constructed and cloned into pTAL destination vector using Golden Gate cloning method. The TALE DNA-binding domain was excised from pTAL using EcoRI and Bam H1 and subsequently subcloned into pcDNA-3.1 mammalian expression vector containing a CMV promoter (Invitrogen). The native TALE activation domain was replaced with four copies of the minimal VP16 transactivation domain, VP64. The VP64 DNA fragment was PCR amplified from plasmid pLenti-EF1a-Backbone (NI) obtained from addgene (http://www.addgene.org/27962) and a SV40 nuclear localization signal added to N-terminus of VP64 using primers NLS_VP64 forward: 5′CCGCGGAGCCCCAAGAAGAAGAGAAAGGTGCTGTCGACGGCCCCC-’3 and VP64 reverse: 5′CTCGAGTCAGTTAATCACATGTCCAGG-′3. The resulting NLS_VP64 PCR product was fused to the C-terminus of TALE protein using Sac II and Xho I restriction enzymes. To construct TBP-TALE, full length TBP (NM_003194.4) was cloned from Jurkat cDNA using forward primer NLS_TBP for: 5′-CCGCGGTCCCAAGAAGAAGAG AAAGGTGGATCAGAACAACAGCCTGC-′3 and reverse primer TBP rev: 5′-CTCGAGTTACGTCGTCTTCCTGAATCCC-’3, and subsequently cloned to pCR 2.1 TOPO vector. Final TALE-TBP constructs were generated by fusing TBP to the C-terminus of TALE constructs using Sac II and Xho I. For detection of TALE constructs, an HA-epitope was fused in-frame to the N-terminus of all TALE constructs. ALL TALE constructs were confirmed by western blot and DNA sequence analysis using primer Seq_TALEN 5-1 for: 5′-CATCGCGCAATGCACTGAC-′3. For the human IL-2 promoter, selection of target sites were chosen based on previously published guidelines [3]–[5], [28] and potential off-targets determined using BLAST analysis. For the human GM-CSF promoter, TALE target sites were selected and potential off-targets were determined using TAL effector nucleotide targeter 2.0 (TALE NT 2.0) software [29]. TALE NT 2.0 assessment of TALE binding sites within the human promoterome indicated that TALE activators scored the highest at their intended target sites.

### Cell Culture and DNA Transfection

Cell lines 293FT, EC9706, MCF-7, Hela, and HepG2 (ATCC) were maintained in Dulbecco’s MEM supplemented with 10% fetal bovine serum and 1% penicillin-streptomycin. HEK293FT cells were supplemented additionally with 1 mM L-glutamine. Jurkat and K562 cell lines (ATCC) were maintained in RPMI supplemented with 10% fetal bovine serum and 1% penicillin-streptomycin. All cell lines were cultured at 37°C with 5% CO_2_. Cell lines were transfected with either Lipofectamine LTX (Invitrogen) or Nucleofector technology (Lonza) as per manufacturer’s instructions. For TALE activation experiments, we transfected all cell lines (150,000 cells per well of a 6-well plate) with the exception of Jurkat cells with 900 ng of empty vector control or TALE-encoding plasmids, in which each TALE plasmid was represented in equal amounts (e.g. two TALEs - 450 ng each or four TALEs –225 ng each) to assess minimal number of TALE activators required for gene activation. For experiments using Jurkat cells, 1x10^6^ cells were transfected with 2 µg of empty vector or TALE-encoding plasmids using equal amounts of each plasmid (e.g. two TALEs –1 µg each or four TALEs –500 ng each). Seventy-two hours post-transfection, cell culture media and cell pellets were collected and stored at −80°C for ELISA analyses and qRT-PCR, respectively.

### Total RNA Isolation and Quantitative RT-PCR

Total RNA was isolated using E.Z.N.A. Total RNA kit (Omega Biotek). Total RNA was reverse transcribed to cDNA using TaqMan Reverse Transcription Reagents (Applied Biosystems). Real Time qPCR was performed using PerfeCTa SYBR Green Master Mix (Quanta Bioscience) using 100 ng of cDNA in 20 µl and amplified using 95°C for 2 minutes followed by 40 cycles of 95°C for 15 seconds, 60°C for 1 minute with data collection after completion of each cycle. The following primer sets were used for qRT-PCR: IL-2 forward primer: 5′-AAACTTTCACTTAAGACCCAGG-′3, IL-2 reverse primer: 5′-GTTGTTTCAGATCCCTTTAGTTCC-′3; GM-CSF forward primer: 5′-ATGTGAATGCCATCCAGGAG-′3, GM-CSF reverse primer: 5′- TGTTTCATTCATCTCAGCAGCA-′3; β-actin forward primer: 5′-ACCAACTGGGACGACATGGAGAAA-′3, β-actin reverse primer: 5′-TTAATGTCACGCACGATTTCCCGC-′3; GAPDH forward primer: 5′- CAATGACCCCTTCATTGACC -′3, GAPDH reverse primer: 5′- TTGATTTTGGAGGGATCTCG-′3. Primer specificity was confirmed by agarose gel electrophoresis and melt curve analysis. Primer amplification efficiency was determined over a dynamic range and only primer sets with 90–110% amplification efficiencies were used in this study. Fold change in IL-2 or GM-CSF expression was calculated using the ΔΔ C_t_ method and expressed relative to empty vector control. IL-2 and GM-CSF experiments were normalized to β-actin or GAPDH, respectively, to control for RNA loading and integrity.

### Enzyme-linked Immunosorbent Assay

Cell culture medias were collected at 72 hr post-transfection and frozen at −80°C for later use. On day of assay, samples were thawed at 37°C and cell culture media was concentrated 30-fold using Amicon ultracel-10 collection units (Millipore). Secreted IL-2 protein was assayed using Quantikine IL-2 ELISA detection kit (R&D Systems) and secreted GM-CSF protein was assayed using Human GM-CSF ELISA Ready-SET-Go! (eBioscience) as per manufacturer’s instructions. To quantify IL-2 and/or GM-CSF protein, a standard curve was plotted as mean absorbance for each standard on the y-axis against the concentration on the x-axis and the best fit line determined by linear regression analysis. For GM-CSF ELISA, the quantity of GM-CSF protein in samples was normalized to empty vector control by background subtraction.

### Chromatin Accessibility using Real-time PCR (CHART-PCR)

CHART-PCR for the IL-2 promoter was performed based on a previously reported method [30], [31]. Briefly, 293FT cell nuclei were isolated using EZ nucleosomal DNA prep kit (Zymo Research) and incubated with 0.5 units of Atlantis DNase I at 42°C for 20 minutes. Nucleosomal DNA purification was carried out using Zymo-Spin IIC columns and eluted in 20 µl of DNA elution buffer or nuclease-free water. A control without DNase I for each sample was included to monitor endonuclease activity and used to calculate accessibility. Isolated genomic DNA (20 ng) was used to perform real-time PCR using PerfeCTa SYBR Green Master Mix (Quanta Bioscience) and PCR primers specific to regions across the IL-2 promoter. The following primer sets were used in the study: Set 1 forward primer: 5′-CTTTTGTATCCCCACCCCCTT-′3, Set 1 reverse primer: 5′-AAACCCCCAAAGACTGACTGA -′3; Set 2 forward primer: 5′-GGGCTAATGTAACAAAGAGGGATT-′3, Set 2 reverse primer: 5′-AACCCATTTTTCCTCTTCTGATGA -′3; Set 3 forward primer: 5′-TATGTAAAACATTTTGACACCCCCA-′3, Set 3 reverse primer: 5′- TGGCAGGAGTTGAGGTTACTG-′3; Set 4 forward primer: 5′-CTACTCACAGTAACCTCAACTCCT-′3, Set 4 reverse primer: 5′-TGTAGAACTTGAAGTAGGTGCACT-′3; GAPDH promoter forward primer: 5′-CGCACGTAGCTCAGGCCTCAAGACC-′3, GAPDH promoter reverse primer: 5′- GGCTGACTGTCGAACAGGAGGAGCA-′3. Primer Set 2 and Set 4 were previously published [31]. GAPDH primer sequences were obtained from Bio-Rad. The amplicon length was kept between 90 and 150 bp. Primer specificity was confirmed by agarose gel electrophoresis and melt curve analysis. Primer amplification efficiency were determined over a linear range and only primer sets with optimal amplification efficiencies were used in this study. Thermocycler conditions were programmed as follows: 95°C/10 min for 1 cycle; 95°C/15 s, 60°C/1 min for 40 cycles and data collection was performed at the end of each cycle. To determine percent accessibility to DNase I digestion, the EpiQ chromatin analysis tool (Bio-Rad) was used to analyze each sample using the following equation: % Accessibility to DNase I = (1 − 2^ΔCt^ ) × 100. To calculate the ΔC_t_ for each sample, the C_t_ value of the DNase I-treated sample were subtracted from the C_t_ value of untreated sample. The human GAPDH promoter (e.g. accessible promoter) was used as an internal control to monitor degree of DNase I digestion and only samples digested similarly were used to determine IL-2 chromatin accessibility.

### Statistical Analysis

To determine statistical significance of TBP-TALE enhancement on gene synergy, statistical analyses were performed using the one-tailed, paired *t*-test. Chromatin analysis was evaluated using the one tailed, Welch’s *t*-test.

## Results

### TBP-TALE can Effectively Activate Expression of the Silenced IL-2 Gene in Non-immune Cells

**Figure 1:**
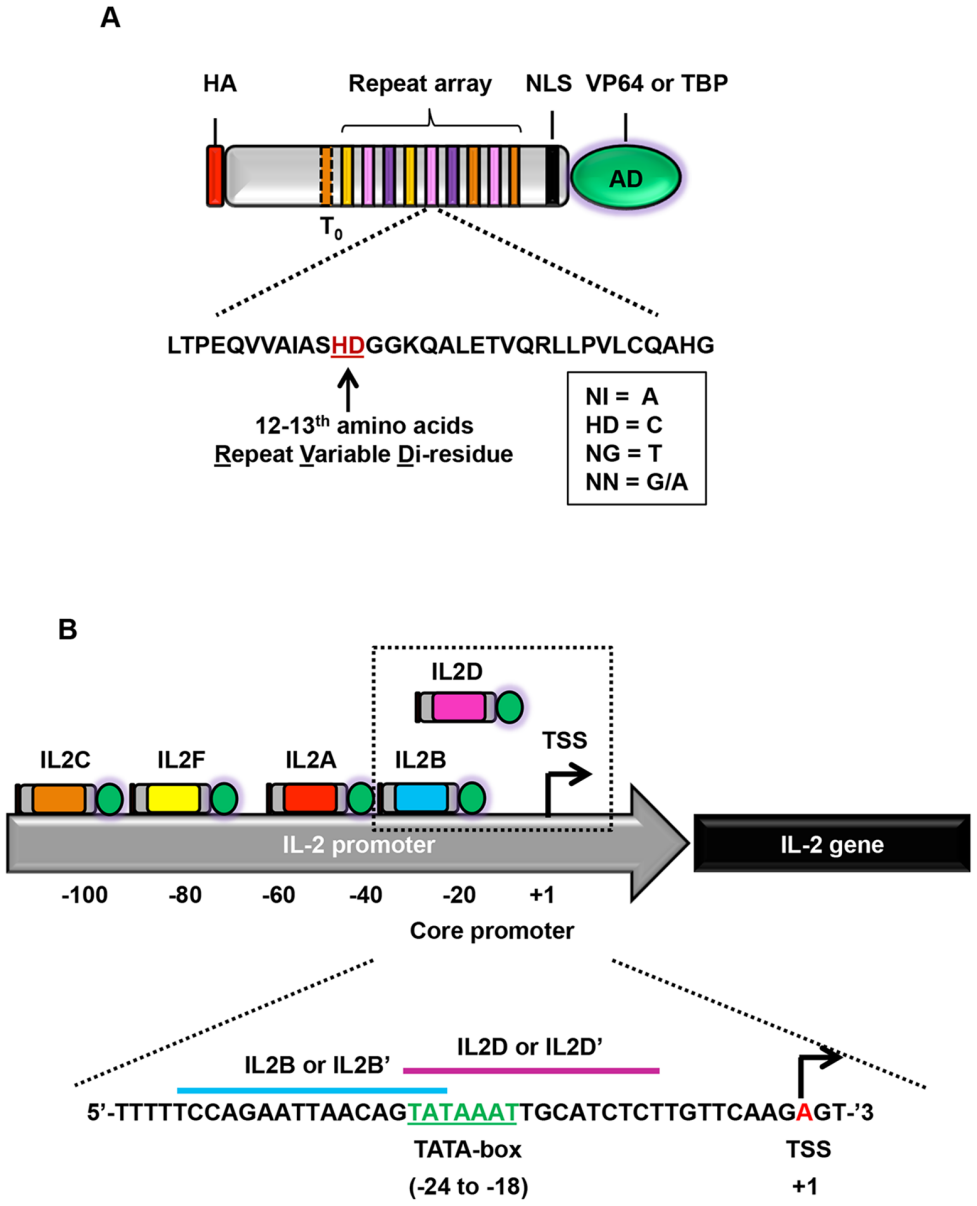
Design of TALE activators targeting the IL-2 promoter. (A) Illustration of engineered TALE construct. HA, hemagglutinin epitope; NLS, SV40 nuclear localization signal; T_0_, recognition of invariant thymine residue; AD, activation domain - VP64 or TATA-box binding protein (TBP). Listed below the construct is the amino acid composition of a typical repeat domain and the RVDs used for DNA recognition. (B) Schematic of the IL-2 promoter (not drawn to scale) and approximate position of each TALE construct relative to the transcriptional start site (TSS). Each TALE is denoted by ‘IL2’ followed by its corresponding alphabet. Enclosed in the dashed-box are TALEs that bind to the TATA-box and their cognate DNA sites are indicated below. All TALEs are fused to VP64 with the exception of TATA-box binding TALEs, which are fused to VP64 (IL2B or IL2D) or TBP (IL2B’ or IL2D’) for comparative analysis. TATA-box is indicated in green. TSS is indicated in red.

The composition of individual components in a typical TALE activator is illustrated in Figure 1A. TALEs were based on the reported N_1_-C_2_ architecture [3], maintained the invariant 5′ thymine, and utilized the conventional RVD code for DNA recognition (Figure 1A). We constructed up to five TALE activators targeting different loci within approximately 100 bp upstream of the transcription start site (TSS) in the IL-2 promoter region (Figure 1B, Table 1). Two of those TALEs, IL2B and IL2D, specifically target the TATA-box (Figure 1B, Table 1). With the exception of TATA-box binding TALEs, all constructs were fused to the potent VP64 transactivation domain; whereas, TALEs directed to the TATA-box were fused to either VP64 (IL2B or IL2D) or TBP (IL2B’ or IL2D’) for comparative analysis. To avoid potential off-target effects, all TALEs were designed to recognize a 15–16 bp DNA target site with regions low in guanine residues (Table 1).

**Table 1:** IL-2 TALE target sites and RVD composition**.**

TALE	DNA Target Site	RVD Sequence	Length (bp)	% NI (A)	% NG (T)	% HD (C)	% NN (G)	Distance from lastRVD to TSS
	(Precluded by ‘T’)							
IL2A	TGACACCCCCATAATA	NG NN NI HD NI HD HD HD HD HD NI NG NI NI NG NI	16	38	18	38	6	−43
IL2B	CCAGAATTAACAGTAT	HD HD NI NN NI NI NG NG NI NI HD NI NN NG NI NG	16	40	30	20	10	−26
IL2C	GTTTTTTCAGACAGGT	NN NG NG NG NG NG NG HD NI NN NI HD NI NN NN NG	16	19	44	13	25	−100
IL2D	ATAAATTGCATCTCT	NI NG NI NI NI NG NG NN HD NI NG HD NG HD NG	15	33	40	20	7	−9
IL2F	GAAAATATGTGTAAT	NN NI NI NI NI NG NI NG NN NG NN NG NI NI NG	15	47	33	0	20	−80

**Figure 2:**
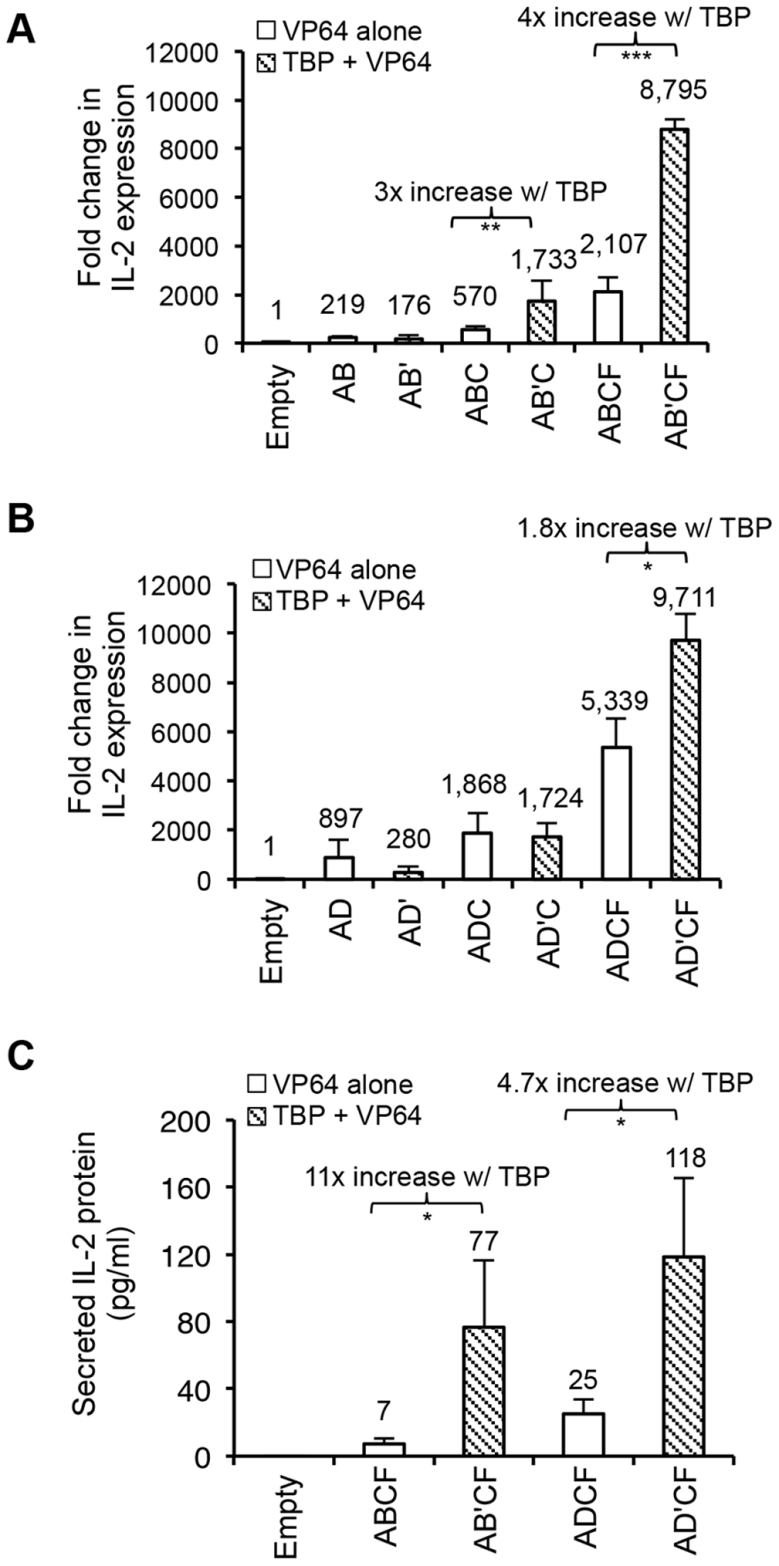
A unique chimeric TBP-TALE drives robust and enhanced IL-2 gene activation. Comparative qRT-PCR analysis of IL-2 gene activation using TBP-TALE and VP64-TALE activators in 293FT cells. (A) TALE-IL2B or (B) TALE-IL2D fused to VP64 (white bars, B or D) or TBP (shaded bars, B’ or D’) was co-transfected with the indicated VP64-TALEs (indicated as A, C, or F) in combinations of two, three or four as described in Methods. Results are shown as fold change in IL-2 gene expression relative to empty vector control. (C) Comparative ELISA analysis of secreted IL-2 protein using the indicated TALE activators. Combinations of four TALE activators were used to induce IL-2 protein expression**.** Data shown is the amount of secreted IL-2 protein in 30-fold concentrated samples. Results shown for qRT-PCR and ELISA are from three independent experiments and error bars are mean +/− SD (n = 3). N.D., not detected. Statistical analysis determined using one-tailed, paired *t*-test (P<0.0005***, P<0.005**, and P<0.05 *).

To test if these TALE constructs could activate the silent IL-2 gene in non-immune cells, we transfected them into 293FT cells using combinations of two, three, or four TALE activators. For comparative analysis of TBP-TALE function, TALE combinations were setup with one of the TATA-box targeting TALEs fused to either VP64 (IL2B or IL2D) or to TBP (IL2B’ or IL2D’) and then used in combination with the indicated VP64-TALE activators (IL2A, IL2C, IL2F). Three days post-transfection, total RNA and cell culture media were collected for qRT-PCR and ELISA quantification of IL-2 transcript and protein, respectively. In agreement with recent reports by others [20], [21], combinations of VP64-TALE activators significantly potentiates their activity in transactivating the expression of IL-2 transcripts (Figure 2A and 2B). The potentiating effect was most obvious in three and four VP64-TALE activator combinations. However, replacing one of the conventional TALE activators (IL2B or IL2D) with a TBP-TALE (IL2B’ or IL2D’) resulted in up to 4-fold enhancement of IL-2 expression in comparison to VP64-TALEs alone, with a maximal IL-2 induction of almost 10,000-fold over that of empty vector control (Figure 2A and 2B). In data not shown, non-transfected cells demonstrated no significant difference in the basal level of IL-2 transcripts when compared with empty vector control indicating that TALE activators and not DNA transfection itself resulted in the transactivation of the IL-2 gene expression. In agreement with the observed increase in IL-2 gene transcripts, the protein form of this cytokine was increased up to 11-fold by the substitution of VP64-TALE with a TBP-TALE when compared to the VP64-TALE activators alone (Figure 2C). Collectively, these results demonstrate that TBP-TALE is a unique activator that can act synergistically with VP64-TALE activators to significantly potentiate the transactivation of the silenced IL-2 promoter in non-immune cells.

### Dynamic TBP-mediated IL-2 Activation Across Diverse Cell Origins

**Figure 3:**
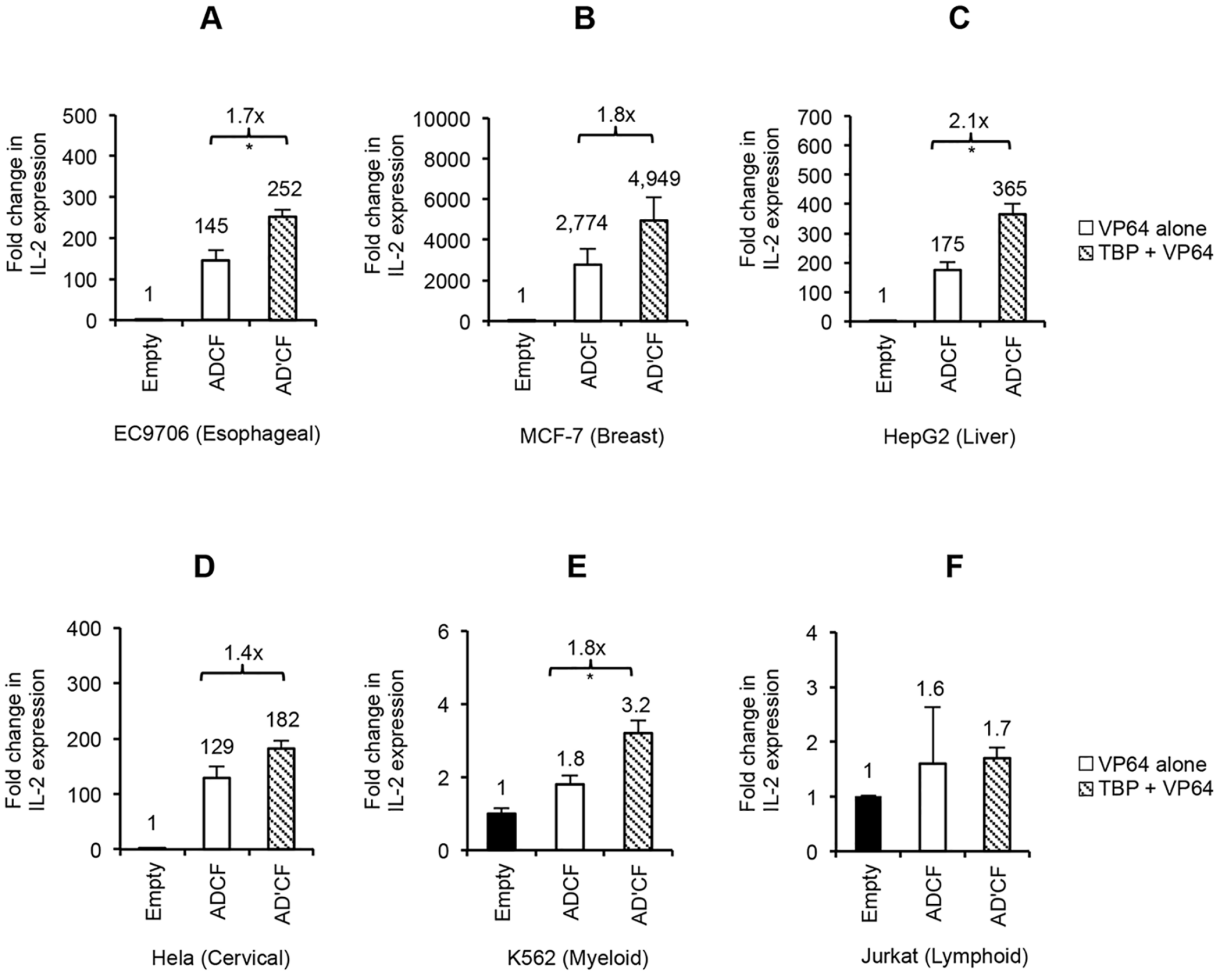
TBP-TALE enhances IL-2 transcriptional activation across diverse cell types. (A–F) Comparative qRT-PCR analysis of IL-2 gene expression in non-immune and immune-related cell lines. Multiple cell lines were transfected with either empty vector control alone, IL2D-VP64 (white bars, D) or IL2D-TBP (shaded bars, D’) plus remaining VP64-TALE activators (A, C, or F). Bar graphs shown are fold change in IL-2 gene expression relative to empty vector control. Below each graph is the identity and type of cell line used in study. EC9706, MCF-7, HepG2, and Hela are non-immune cell lines of epithelial origin. Jurkat and K562 are immune-related cell lines of hematopoietic origin. Results shown are of two independent experiments and error bars are mean +/− SD (n = 2). Statistical analysis determined using one-tailed, paired *t*-test (P<0.05 *).

**Figure 4:**
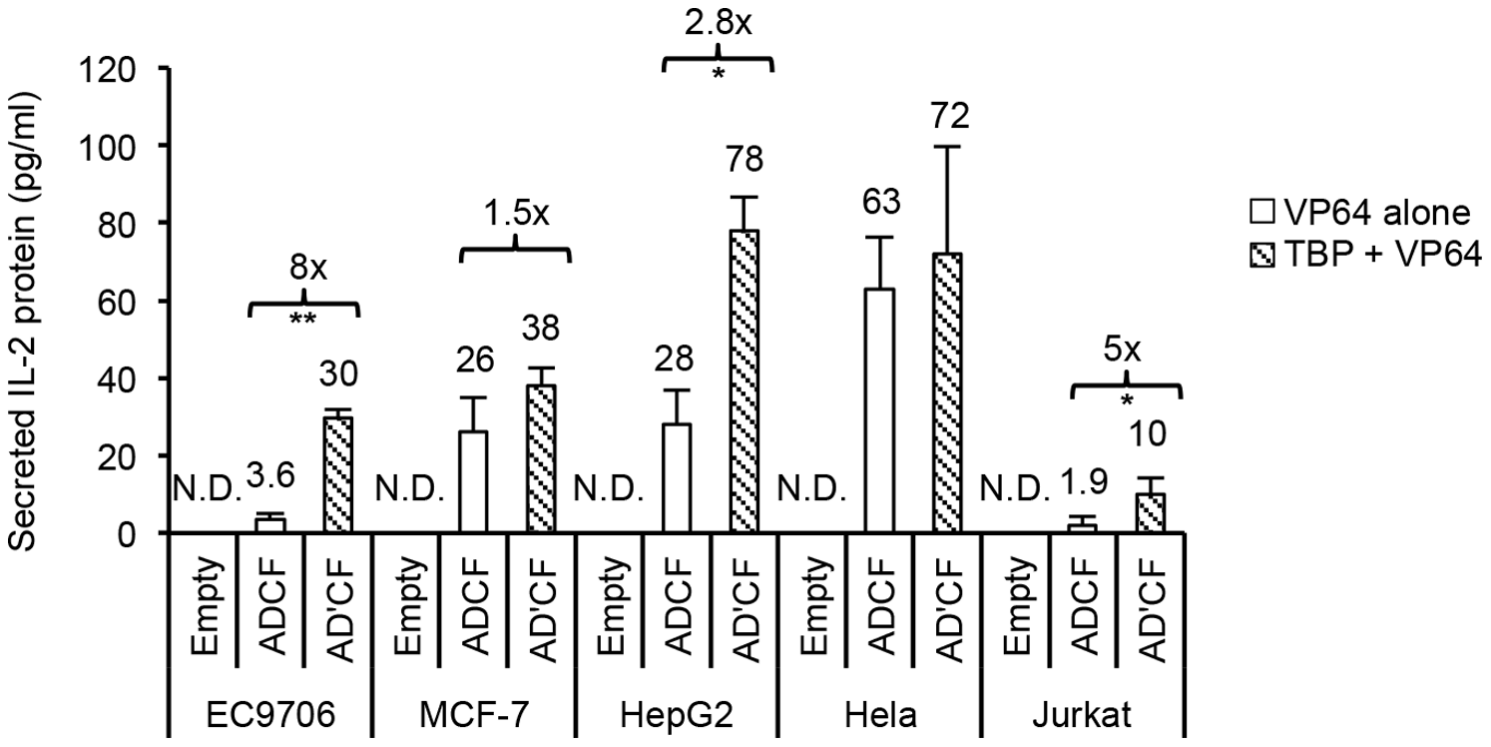
Detection of IL-2 protein confirms potentiation capability of TBP-TALE. Comparative ELISA analysis of secreted IL-2 protein confirms potentiation capability of TBP-TALE. Multiple cell lines were transfected with either empty vector control alone, IL2D-VP64 (white bars, D) or IL2D-TBP (shaded bars, D’) plus remaining VP64-TALE activators (A, C, and F). Data shown indicates the amount of secreted IL-2 protein in 30-fold concentrated samples. N.D. indicates ‘not detected’. Results shown are of two independent experiments (error bars, mean +/− SD, n = 2). Statistical analysis determined using one-tailed, paired *t*-test (P<0.005 **, P<0.05 *).

Current studies employing TALE activators in diverse cell origins are limited. The activation potential of TALEs in the context of cell-type specific chromatin environments, which can influence the level of achievable gene activation [32], has not been fully evaluated. Having established the potency of TBP-TALE in synergizing with other VP64-TALE activators to switch on the silenced IL-2 gene in 293FT cells, we next sought to probe its activity in this regard in the context of either non-immune cells of epithelial origin or immune-related cell lines of hematopoietic origin. Based on reports by others [20], [21] and our experiments done in 293FT, using four or more TALE activators provides the highest level of synergistic gene activation. Therefore, we tested the ability of four TALE activators to switch on silenced IL-2 gene expression in various cell lines. To do so, we compared the function of our most active TATA-box binding TALE fused to either VP64 (IL2D) or to TBP (IL2D’) and then used either one in combination with the remaining VP64-TALEs (IL2A, IL2C, and IL2F). Consistent with what we observed in 293FT cells, we found that IL2D-TBP (IL2D’) coupled with three additional VP64-TALE activators enhanced synergy up to ∼2-fold in all the non-immune cells tested, as well as in K562 cells of myeloid origin (Figure 3A-3E). Interestingly, in the only cell line of lymphoid origin (Jurkat), low basal levels of IL-2 were detectable and enhancement of synergistic gene activation was not seen (Figure 3F). Consistent with the observed increase in IL-2 transcription, secreted IL-2 protein was detectable in the cell culture media of all cell lines tested using the four combinations of TALE activators (Figure 4). The enhancement effect by IL2D-TBP in combination with remaining VP64-TALEs was more pronounced at the protein level, with up to 8-fold increase in some cells when compared with VP64-TALEs alone. Unexpectedly, despite lack of enhancement effect on the transcriptional level in Jurkat cells, IL2D-TBP plus remaining VP64-TALEs led to a relatively low but measurable level of IL-2 protein in the medium (Figure 4). Collectively, we demonstrate that the combined action of a single TBP-TALE with additional VP64-TALEs leads to enhanced induction of IL-2 gene expression across a diverse setting of non-immune cells, with moderate induction of the gene expression in cells of lymphoid origin which appears to have occurred at the translational level.

### Recapitulation of TBP-mediated Enhanced Synergistic Effect on the GM-CSF Promoter

**Figure 5:**
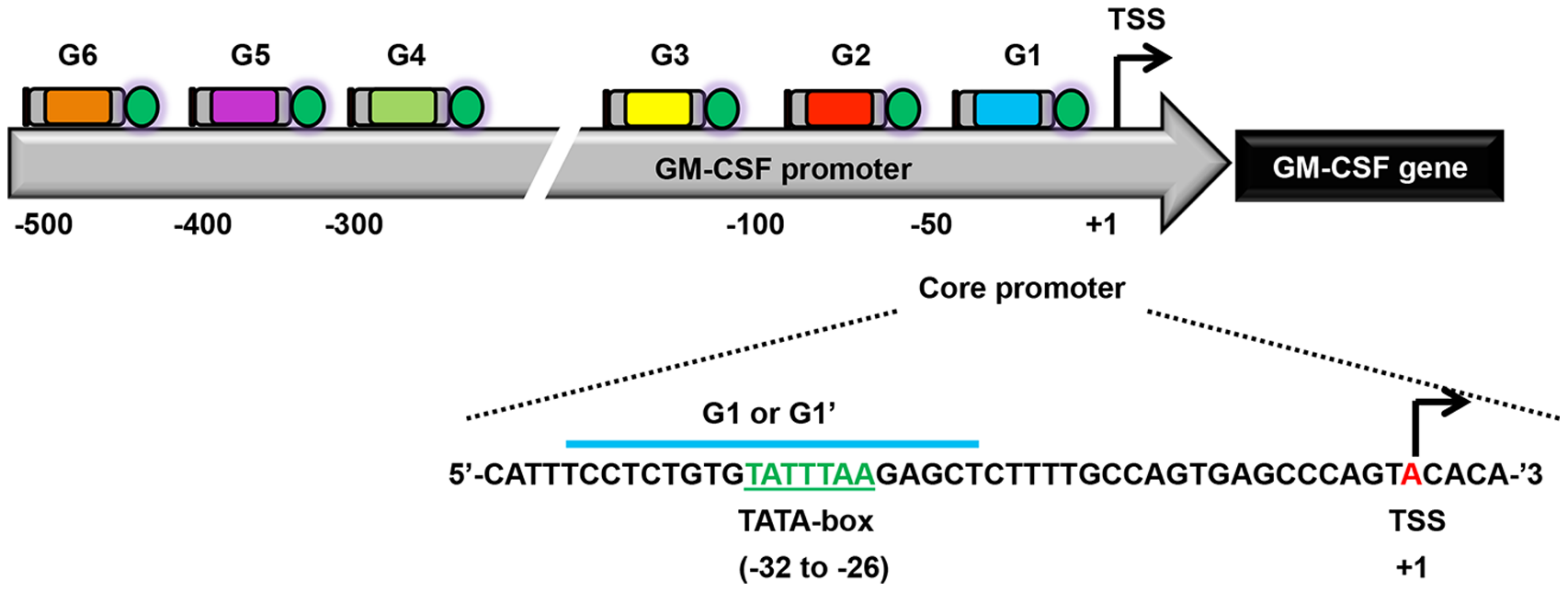
Targeting of the silent GM-CSF promoter using TALE activators. A schematic of the GM-CSF promoter and approximate location of GM-CSF TALE activators relative to the TSS (not drawn to scale). TALE identity is denoted above each TALE illustration. GM-CSF TALEs target within 500 bp of the TSS. TALE-G1 binds across the TATA-box and its cognate DNA sequence is indicated below. All TALEs are fused to VP64 except for TALE-G1, which is fused to VP64 (denoted as G1) or TBP (denoted as G1’) for comparative analysis. The TATA-box is highlighted in green and its position relative to the TSS (red) indicated below.

**Figure 6:**
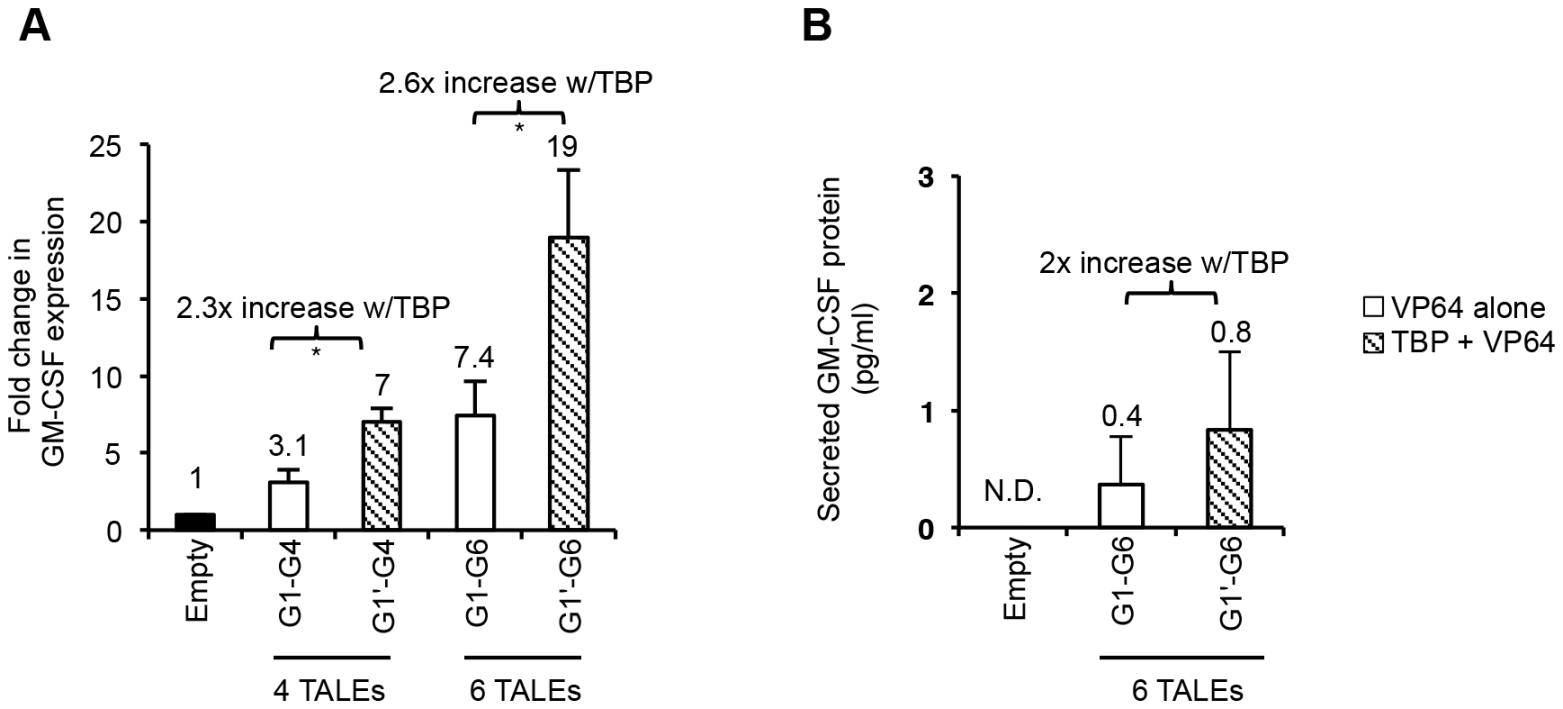
TBP-mediated enhancement of GM-CSF activation. (A) Quantitative RT-PCR analysis of GM-CSF gene expression in 293FT cells. 293FT cells were transfected with either empty vector control alone, G1-VP64 (white bars, G1) or G1-TBP (shaded bars, G1’) in combination with indicated GM-CSF specific VP64-TALE activators. Results are shown as fold change in GM-CSF gene expression relative to empty vector control. (B) GM-CSF ELISA analysis of TALE activity in 293FT cells. Results shown are the amount of secreted GM-CSF protein in a 30-fold concentrated sample normalized to empty vector control by background subtraction. ND indicates ‘not detected’. Results shown are of three independent experiments (error bars, mean +/−SD, n = 3). Statistical analysis determined using one-tailed, paired *t*-test (P<0.05*).

To further demonstrate TALE-mediated activation on silenced immune gene expression, we targeted a second immunomodulatory gene, granulocyte-macrophage colony-stimulating factor (GM-CSF). Similar to the IL-2 promoter, the regulatory region of the *GM-CSF* gene is only activated during proper TCR signaling in T-cells or with immunostimulatory agents in fibroblast or endothelial cells [33]–[36]. As recent reports have shown that as many as six TALE activators may be required to potently drive gene expression [20], [21], we designed up to six TALEs to target various positions along the GM-CSF promoter spanning an area ∼500 bp upstream of the TSS (Figure 5). GM-CSF TALEs were designed in a similar way to those of IL-2, to recognize 15–20 bp target sites with minimal guanine content as shown in Table 2. In accord with IL-2 specific TALEs, G1-TBP (G1’) in combination with multiple GM-CSF specific VP64-TALE activators resulted in more than 2-fold enhancement of gene expression over that from VP64-TALEs alone, when using both four and six activator combinations in 293FT cells (Figure 6A). In fact, the inclusion of G1-TBP (G1’) plus three additional VP64-TALEs (G2, G3, and G4) worked just as well as using all six VP64-TALEs (G1-G6). The elevated gene transcription using the combination of G1-TBP (G1’) with additional VP64-TALEs led to the release of GM-CSF protein at a level readily detectable in the culture medium of 293FT cells, in which this gene is otherwise totally silenced (Figure 6B). Taken together, these data suggest that TBP-TALE represents a novel class of potent activators that may be applied to many silenced mammalian genes to turn on their expression in a synergistic way when used in combination with VP64-TALE activators.

**Table 2:** GM-CSF TALE target sites and RVD composition.

TALE	DNA Target Site	RVD Sequence	Length (bp)	% NI (A)	% NG (T)	% HD (C)	% NN (G)	Distance from lastRVD to TSS
	(Precluded by ‘T’)							
G1	CCTCTGTGTATTTAAGAGCT	HD HD NG HD NG NN NG NN NG NI NG NG NG NI NI NN NI NN HD NG	20	20	40	20	20	−19
G2	AGTTCCCCCGCCTCCCT	NI NN NG NG HD HD HD HD HD NN HD HD NG HD HD HD NG	17	6	24	58	12	−69
G3	GAAAACCCCCAAGCCT	NN NI NI NI NI HD HD HD HD HD NI NI NN HD HD NG	16	38	6	44	12	−157
G4	CCAAACTGTGCCCCT	HD HD NI NI NI HD NG NN NG NN HD HD HD HD NG	15	20	20	47	13	−280
G5	AGCCTGCCCAAAGGCCCCT	NI NN HD HD NG NN HD HD HD NI NI NI NN NN HD HD HD HD NG	19	21	11	47	21	−370
G6	CCCCTCACACTCAAGTCT	HD HD HD HD NG HD NI HD NI HD NG HD NI NI NN NG HD NG	18	22	22	50	6	−467

### TALE Activators Promote Nucleosome Displacement upon the Silenced Gene Promoter

Next, we sought to elucidate the mechanism(s) by which TBP-TALE in combination with VP64-TALEs robustly activated silent IL-2 gene expression in non-immune cells. We wanted to know if these artificially constructed TALE activators were able to activate the IL-2 gene expression by directly binding to the chromatin as reported by others [21] or if chromatin remodeling was necessary. Within naïve T-cells, the IL-2 chromatin architecture is maintained in an inaccessible state, formed by nucleosome accumulation within the proximal promoter region, which masks TCR-specific response elements [30], [31], [37]–[41]. Consequently, nucleosome masking of these elements prevents TFs from binding to their cognate target sites and inhibits IL-2 gene transcription. However, upon T-cell stimulation, the IL-2 promoter is remodeled to an accessible state, which accommodates multiple TFs binding such as NFAT, AP-1, Oct-1 and NF-κB family members [36], [37], [40], [42]–[44]. Thus, chromatin remodeling accompanied by specific TFs is required to initiate IL-2 gene expression.

**Figure 7:**
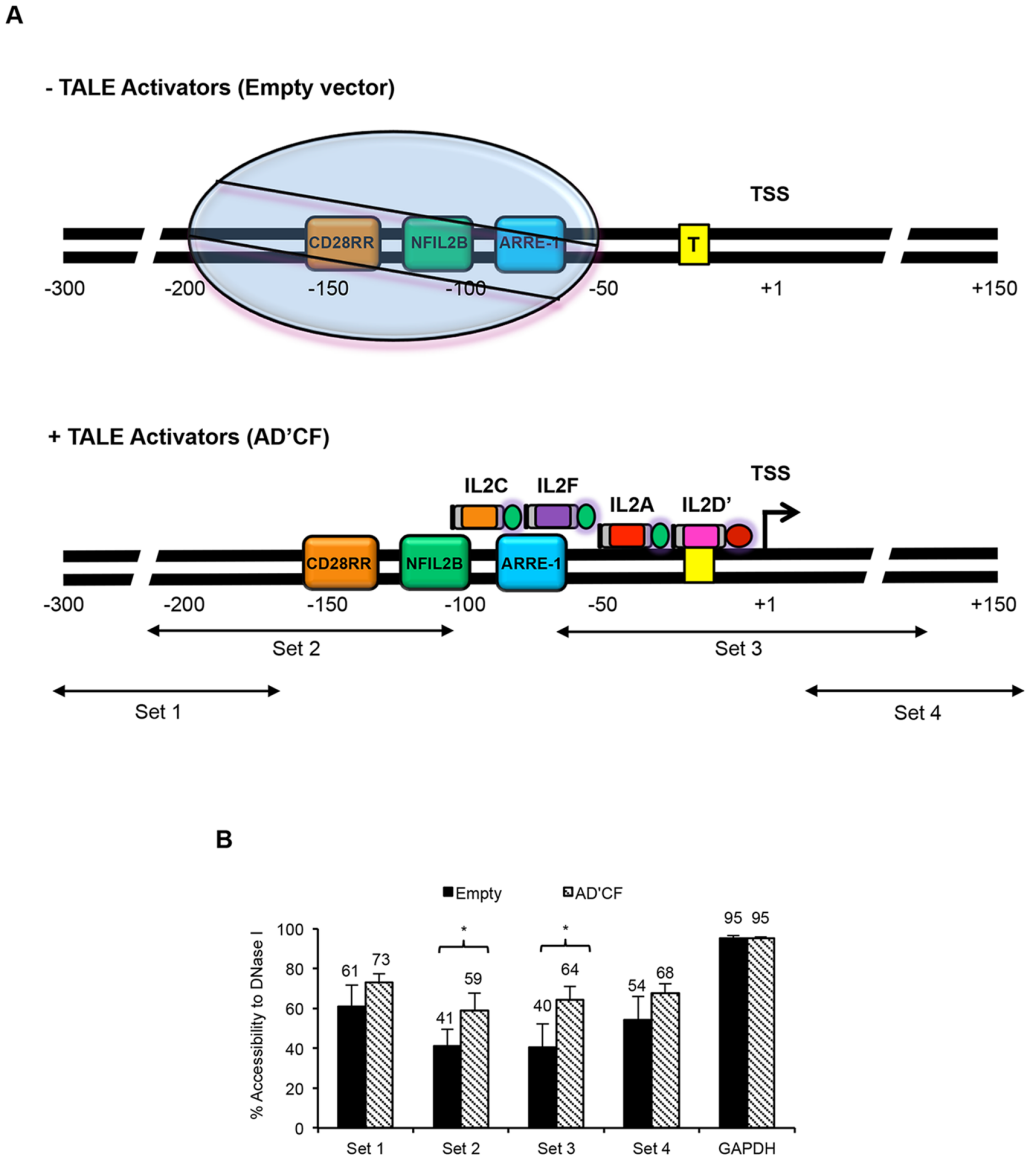
TALEs facilitate displacement of a positioned nucleosome on the IL-2 promoter. (A) Proposed TALE mechanism upon binding to the IL-2 promoter (not drawn to scale). Top panel is an illustration of the proposed chromatin structure of the IL-2 promoter in the absence of TALE activators. The approximate location of response elements adapted from previously published data [31]. Circle represents a positioned nucleosome located approximately 60 to 200 bp upstream of the TSS [31]. Boxed enclosed ‘T’ represents the TATA box. Bottom panel indicates location of TALE activators relative to TSS and proposed mechanism of action on the IL-2 promoter. IL-2 specific primers were used to probe various regions across the IL-2 promoter. Arrows indicate the approximate location of each region amplified by their corresponding primer set. All TALE activators were fused to VP64 (activation domain colored green) with the exception of IL2D’, which was fused to TBP (activation domain colored red). (B) Results of chromatin analysis using CHART-PCR. Percent accessibility was calculated as described in Methods and plotted for both empty vector control (black bars) and TALE activators (shaded bars). GAPDH used as internal control to monitor DNase I digestion. Results shown are of three independent experiments (error bars, mean +/− SD, n = 3). Statistical analysis was determined using one tailed, Welch’s *t*-test (P<0.05 *).

Previous studies have shown that resting T-cells can position a distinct nucleosome between 60 to 200 bp upstream of the TSS and renders the IL-2 promoter inactive [31]. During T-cell activation, this distinct nucleosome is subsequently displaced and accompanies increased DNase I hypersensitivity followed by ensuing IL-2 gene activation [31]. To investigate the IL-2 promoter chromatin architecture in the presence of TBP-TALE and VP64-TALE activators in non-immune cells, we co-transfected 293FT cells with the most active combination of TALE activators (AD’CF) or with empty vector control followed by CHART-PCR (short for, **Ch**romatin **A**ccessibility **R**eal **T**ime PCR) as previously described [30], [31]. CHART-PCR quantitatively measures the accessibility of a particular DNA region to DNase I cleavage as measured by real time qPCR. In concept, regions of open or relaxed chromatin DNA are more sensitive to DNase I cleavage, whereas regions of closed chromatin DNA are resistant to cleavage. Hence, we hypothesized that in the presence of TALE activators, the IL-2 promoter would be more sensitive to DNase I cleavage than that of empty vector control. As shown in Figure 7A, a series of primers were designed to probe the accessibility status of different loci within the IL-2 promoter region. The results from the CHART-PCR revealed that the combination of AD’CF TALEs but not empty vector control increased DNase I hypersensitivity across the IL-2 promoter with a significant increase observed in regions probed by primer sets 2 and 3 (Figure 7B). Interestingly, both primer sets amplified genomic regions either within the vicinity of a distinctly positioned nucleosome found in resting T-cells [31] or within the TSS (Figure 7A and 7B). Together, these data demonstrate that TBP-TALE together with VP64-TALEs have either displaced or repositioned the nucleosome outside the detection range to allow for exclusive access of TALE activators to the IL-2 promoter regulatory regions, which controls gene activation. In addition, these data are consistent with previous reports describing the activated IL-2 promoter state in stimulated T-cells [30], [31] and thus suggests that non-immune cells are subjected to a similar mechanism of regulation and chromatin configuration.

## Discussion

TALE technology holds great promise in serving as a useful tool to decipher the functionality of genetic elements and in serving as a means to selectively switch on or off genes for therapeutic purpose. In the effort to selectively switch on gene expression using TALE technology, recent studies from two independent laboratories have demonstrated that robust and synergistic gene activation can be achieved using multiple TALE activators [20], [21]. Here we report that this synergistic effect could be further potentiated by up to 11-fold with a novel class of TALE-based activators, TBP-TALE. We demonstrated the potentiation capability of TBP-TALEs using two classical examples of silent, cell-type restricted genes, IL-2 and GM-CSF, in diverse cell lines at both the transcriptional and translational level. These unique TBP-based activators seem to function synergistically with the conventional VP64-TALEs on both genes and on a variety of cell lines that have been tested, indicating their universal potentiation activity in a diverse intracellular environment. The demonstrated potency of TBP-TALEs in synergizing with other VP64 activators to selectively switch on the expression of immunoregulatory genes such as IL-2 and GM-CSF has direct implications for targeted cancer immunotherapy and other similar applications.

Although our studies, together with two recent publications [20], [21], have clearly shown that multiple VP64-TALE activators alone or in combination with TBP-TALE can act synergistically to switch on silenced genes, the detailed mechanism of such a synergistic action has not been fully elucidated. One report has suggested that TALE activators can be designed with negligible regard for chromatin structure [21]. However, based on extensive characterization of both the IL-2 and GM-CSF promoters, it is possible that their proximal and/or core promoter regions require extensive chromatin remodeling to activate gene expression [30], [31], [37], [38], [40], [45], [46]. In supporting this notion, our data indicate that the robust TALE-mediated activation on the IL-2 promoter was in part due to altered chromatin accessibility possibly attributed to the action of VP64-TALE activators in collaboration with TBP-TALE directed initiation of transcription. As transcriptional initiation begins with the recruitment of TBP, it is plausible that targeted binding of TBP-TALE to the TATA box in cytokine gene promoters can bypass this rate limiting initial step of transcription for a silenced gene and facilitate mechanisms directed by VP64-TALEs such as, displacing or repositioning the nucleosome to allow for exclusive access of TALE activators to the promoter regulatory regions.

In addition, other cooperative interactions between TBP and VP64-TALEs may also contribute to their synergistic activation on the cytokine genes. Potent transcriptional activators like VP16, in which our study uses four copies of it (VP64), interact with multiple components of the basal transcriptional machinery and more importantly with TBP to drive initiation of transcription. Specifically, the interaction between VP16 has been shown to stabilize TBP to the TATA-box and synergistically activate gene expression [22], [27], [47], [48]. Hence, it is likely that VP64-TALEs stabilize TBP-TALE to cytokine gene promoters through cooperative interactions and this led to synergistic activation of cytokine genes. Additional data to support this notion is supported by a few instances in TBP-TALE experiments. As shown in Figures 2A and 2B, two TALE combinations using TBP-TALE demonstated an apparent reduction in IL-2 gene expression and appreciable levels of enhanced synergy at the transcriptional level was only demonstrated when using multiple VP64-TALE activators coupled with TBP-TALE. We speculate that in these instances, TBP-TALE binding and subsequent bending of the DNA, a natural function of TBP, directly influenced its cooperative interaction with the neighboring VP64-TALE (IL2A) leading to a reduction in gene activation. However, as the number of VP64-TALEs increased, this stabilized TBP-TALE to the gene promoter leading to enhanced IL-2 gene activation. Taken together, we speculate that cooperative and reciprocal interactions between TBP-TALE and VP64-TALEs enables them to effectively initiate robust transcriptional activation of silenced genes.

Additionally, our data seems to suggest that the position of TBP-TALE relative to neighboring VP64-TALEs affects its potentiation capability on gene activation. For example, the data in Figure 2A and 2B demonstrate that TBP-TALEs differentially influence the extent of synergistic effect, with IL2B-TBP enhancing synergy two times more efficiently than IL2D-TBP. The distance between IL2B-TBP and an adjacently located VP64-TALE (IL2A-VP64) is 5 bp in contrast to 20 bp for IL2D-TBP, with the former enhancing gene activation up to 4-fold and the latter only ∼2-fold. A recent study reports a similar position effect on gene activation between adjacent DNA-bound transcription factors [49]. Furthermore, our data suggests that the ability of TBP to synergize gene expression with VP64 activation domains is also sensitive to the relative position of neighboring activators, which is also supported by other studies in the literature [48]. Thus, it is plausible that the close proximity of IL2B-TBP to IL2A-VP64 formed a more stable complex than that of IL2D-TBP and IL2A-VP64 which influenced the degree of synergy when combined with additional VP64-TALE activators. As for the GM-CSF TALEs, G1-TBP (G1’) was positioned nearly ∼30 bp relative to G2-VP64 (G2) and supported similar enhancement of synergy as demonstrated by IL2D-TBP. Collectively, we speculate that the ability of TBP-TALEs to synergistically activate and potentiate gene activation may be attributed to mutual contributions from natural TBP function, cooperative interactions amongst TALEs, and positioning of TBP-TALE relative to neighboring VP64-TALE activators.

Future refinement of TALE technology should strive to interrogate post-transcriptional mechanisms mutually important for overall success of gene activation technologies such as, 3′-UTR regulation, cryptic 5′-UTR signals, alternative spliced variants, negative feedback loop mechanisms, and protein stability. As for the cytokines in this study, both are subjected to various post-transcriptional mechanisms such as mRNA stability (e.g. AU-rich regions), alternative splicing to produce dominant negative variants, and cryptic 5′UTR elements (e.g. premature stop codon), all of which may function to destabilize and/or fine tune gene expression [50]–[54]. The potential role of TALE activators on post-transcriptional regulation of gene expression may partly explain the discrepancy between the level of transcripts and protein products in several occasions in our experiments. Further experimentation should work to test these parameters as they may shed light on novel ways to enhance the translational outcome of gene activation while taking into consideration gene-specific epigenetic and post-transcriptional mechanisms which influence gene activation beyond the realm of transcriptional initiation. Future studies will strive to further develop our TALE activators to have diverse functions while exploring post-transcriptional mechanisms that potentiate robust gene activation.
